# Sociodemographic Characteristics and Inadequate Usual Sources of Healthcare in a National Sample of US Refugees

**DOI:** 10.3390/ijerph19127234

**Published:** 2022-06-13

**Authors:** Kyle J. Baumann, Tilahun Adera

**Affiliations:** 1 Division of Epidemiology, Department of Family Medicine and Population Health, Virginia Commonwealth University School of Medicine, Richmond, VA 23298, USA; tilahun.adera@vcuhealth.org; 2 Department of Medicine, University of Minnesota Medical School, Minneapolis, MN 55455, USA; 3 Department of Pediatrics, University of Minnesota Medical School, Minneapolis, MN 55455, USA

**Keywords:** refugee, usual source of care, healthcare access, health insurance coverage

## Abstract

Introduction: Refugees resettled into the United States (US) face challenges in accessing adequate healthcare. Knowledge of demographic and social characteristics related to healthcare access among refugees is scarce. This study examines potential sociodemographic predictors of inadequate usual sources of care (USCs)—one key component of healthcare access—within the US refugee population. Methods: The 2016 Annual Survey of Refugees (ASR) involving 4037 refugees resettled into the US served as the data source for this study. Inadequate USC was defined as a USC that was neither a private healthcare provider nor a health clinic. We used multiple binary logistic regression methods to identify sociodemographic predictors of inadequate USCs. In addition, we used multinomial logistic regression to further assess predictors of inadequate USCs with a particular focus on severely deficit USCs (i.e., emergency department dependence and USC absence). Results: Refugees with interrupted healthcare coverage were more likely to have an inadequate USC. Refugees who were young (age 10–19), resettled into the western region of the US, and highly educated were less likely to have an inadequate USC. Refugees with an education level higher than secondary had a significantly lower likelihood of having a severely deficient USC, while refugees with interrupted healthcare were more than twice as likely to have a severely deficient USC. Conclusions: Considering these results alongside our previous healthcare coverage findings provides a more comprehensive understanding of sociodemographic predictors of poor healthcare access among refugees resettled into the US. This improved understanding has the potential to assist early refugee contacts toward more effective healthcare resource allocation and aid policymakers attempting to improve programs linked to refugee healthcare access.

## 1. Introduction

The full experience of refugee resettlement is often marked by psychological and physical disturbance [1,2,3]. Although the health needs of refugees are well documented, many refugees do not receive appropriate healthcare [4,5]. A variety of barriers to healthcare access exist for the refugee. These barriers include cultural, economic, educational, geographic, and linguistic barriers [4,5]. Amidst a growing understanding of these access barriers and subsequent experienced disparities, many questions remain regarding the primary and emergency care access of US refugees [4].

Many variables affect overall healthcare access, including health insurance coverage, usual source of healthcare (USC), and visiting a healthcare provider within the past year [6]. The pairing of health insurance coverage and a USC has been shown to synergistically increase healthcare access [7]. Between the two, having a USC is a clearer and more powerful predictor of healthcare access [7]. In our prior article, we studied and discussed predictors of interrupted healthcare coverage among US refugees [8]. USCs will now be considered.

Although there is no validated definition of a USC, a simple and sufficient definition is: a place—outside emergent, acute circumstances—where a person usually goes when sick [9]. Having an adequate USC has been shown to improve healthcare access [7]. More specifically, having an adequate USC fosters access to appropriate care at the appropriate place and time [7]. Researchers have demonstrated an association between having an adequate USC and the following: decreased use of emergency services, greater use of preventative services, decreased engagement in risky behaviors, and increased patient satisfaction [10,11]; more positive reports of patient-centered communication [12]; improved control of chronic diseases such as hypertension, diabetes, and hypercholesterolemia [11]; decreased probability of inpatient admissions and readmissions; decreased expenditures on emergency department visits for physical ailments, behavioral health inpatient admissions, and 30-day readmissions, and improved continuity for vulnerable populations [9]. Overall, having an adequate USC not only improves healthcare access, but also healthcare quality and health outcomes.

A greater understanding of the factors affecting healthcare access for refugees is needed to guide public health action and healthcare policy. To our knowledge, socio-demographic predictors of USCs among refugees living in the US have not been comprehensively analyzed using credible data from a national sample of refugees. In this study, we further examine potential sociodemographic predictors of inadequate USCs—a key component of healthcare access—among refugees living in the US using national data from the 2016 Annual Survey of Refugees (ASR).

When using the terms inadequate and deficient throughout this manuscript, we are strictly characterizing the refugee’s source of care, and not the refugee. The purpose of this work is to illuminate the healthcare access disparities faced by refugees—a diverse group of people marked by remarkable strength and resilience—with the goal of improving their resettlement experience in the US.

## 2. Methods

### 2.1. Data Collection

The 2016 ASR data collected by the Office of Refugee Resettlement (ORR) were used for the analysis. The ASR’s cross-sectional dataset from a national sample of refugees resettled into the US focuses on the first five years after refugee resettlement. The ASR is the only credible source of US data on the progress of integration and self-sufficiency for refugees resettled into the US [13,14]. The sample population was defined as refugees who entered the US between fiscal year 2011 and fiscal year 2015, and were 16 years-of-age or older at the time of the 2016 survey interview [14].

The ASR survey design has previously been described in detail [8,14]. Using a stratified probability design with person-level cluster sampling, households, and then persons within each household, were selected for inclusion. Proportionate stratified sampling was also used to most closely represent the actual population of refugees resettled into the US, considering age, gender, year of arrival, geographical region, native language, and household size. A replicated sample design was used to offset the suspected high survey nonresponse rate often seen in the previous ASRs. Survey materials translated into sixteen non-English languages, with one Chaldean interpreter, were used for survey administration. These seventeen non-English languages represented approximately 77% of the overall 2011–2015 refugee population [14]. Trained interviewers gathered information from each household’s principal applicant by phone, utilizing proxy reporting for all other eligible refugees within the same household.

### 2.2. Measures

Data for the outcome variable of an inadequate USC were collected using the following survey question: What is the refugee’s usual source of medical care? The answer options were private physician, emergency room at a hospital, health clinic, folk healer, other, no regular source, don’t know, and refused. New USC variables were created with the following two groupings: (1) adequate USC; (2) inadequate USC. The adequate USC sub-group included private provider and health clinic. The inadequate USC sub-group included emergency room at a hospital, no regular source, folk healer, and other. Refugees who either reported not knowing their usual source of healthcare or refused to answer were excluded from the analysis.

In addition, the inadequate USC outcome was further split into the following two sub-categories: severely deficient USC, and deficient USC. The severely deficient USC category included emergency room at a hospital and no regular source. The deficient USC category included folk healer and other. This subcategorization allowed us to further tease out predictors of the two most concerning reported sources of healthcare: the emergency room and no healthcare source.

For the assessment of predictors related to USCs, potential predictor variables were chosen from the ASR data based on the current USC literature. The potential predictor variables selected were gender, age, citizenship, US region of resettlement, year of resettlement, presence of a debilitating chronic condition, current English proficiency, highest degree obtained prior to resettlement, number of current jobs, marital status, and healthcare coverage status.

### 2.3. Analysis

The frequency and percentage of each of the two USC categories for the sub-groups of the ten potential predictor variables were determined. Unweighted frequencies and weighted percentages according to the ASR person-level weighting scheme were reported. First, the association between USC and each predictor variable was assessed using a chi-square test. The weighted percentages of the two categories were then compared to determine the nature of the association for each predictor variable.

Second, a multiple logistic regression model was developed. Ordinal logistic regression was not used due to failure to satisfy the proportional odds assumption. Eleven predictor variables were originally included in the model. The variable citizenship was left out of the final statistical model due to its overall negative effect on the other independent variables and low cell counts when included. A combination of the Akaike information criterion (AIC) value, Bayesian information criterion (BIC) value, and overall influence on the final model were used to assess the model fitness of the predictor variables with non-statistically significant associations among all subgroups. To note, the AIC and BIC values were utilized without full assessment of the normal residue distribution. Ultimately, the remaining ten potential predictors were included in the model.

Using an a priori approach, one interaction was determined reasonable for the consideration of inclusion in the model. The interaction included the two predictor variables of healthcare coverage status and education level prior to resettlement. When the interaction was added to the model, it was not found to be statistically significant (*p* = 0.48). Therefore, the interaction term was not included in the final model. A simple correlation analysis including all predictor variables was performed. None of the pairs of predictor variables had a correlation greater than ρ = 0.6, suggesting an absence of multicollinearity within the model. To examine whether an association existed between each predictor variable and having an inadequate USC, crude odds ratios were obtained using simple logistic regression for each predictor variable. Next, the multiple logistic regression model was used to obtain adjusted odds ratios for each predictor variable. The regression model provided the likelihood of having an inadequate USC compared to an adequate USC for each predictor variable after controlling for the nine covariates included in the model. The crude and adjusted odd ratios were then compared to assess the confounding factors. To complete the trichotomous USC outcome analysis, using multinomial multiple logistic regression, the severely deficient USC and deficient USC sub-groupings were compared to the adequate USC grouping using the same ten predictors.

A significance value of α = 0.05 was used throughout the entire study. SAS version 9.4 and SAS University statistical software were utilized to perform the analysis (Copyright © 2019 SAS Institute Inc. SAS and all other SAS Institute Inc. product or service names are registered trademarks or trademarks of SAS Institute Inc., Cary, NC, USA). The use of data from the 2016 Annual Survey of Refugees public dataset in this study was reviewed and approved by the Edward Via College of Osteopathic Medicine (VCOM) IRB. Since the study does not meet the regulatory criteria for human subject research, the VCOM IRB concluded that full review and approval were unnecessary.

## 3. Results

Data were collected from 4037 eligible refugees. The sample consisted of slightly more males than females (54% vs. 46%), and a young, predominantly working-age population, with more than 80% under the age of 50. The majority of participants were married and currently employed at the time of the survey. Of the 4037 refugees, 1499 (37.13%) reported an adequate USC and 1233 (30.54%) reported an inadequate USC. To note, 1305 (32.33%) refugees could not or chose not to provide an appropriate answer to the USC question. The descriptive statistics for the USC analysis are displayed in Table 1. The *p*-values are derived from the inadequate USC chi-square analysis.

The results of the binary crude logistic regression and binary multiple logistic regression models comparing inadequate USCs with adequate USCs can be found in Table 2. Prior to adjustment, male refugees were 1.29 times more likely to have an inadequate USC compared to female refugees (95% CI: 1.03, 1.62). Upon covariate adjustment, the association remained similar, although not statistically significant (Adjusted OR: 1.23 [0.99, 1.52]). Refugees 10–19 years old were 36% less likely to have an inadequate USC compared to refugees 20–49 years old before (Crude OR: 0.64 [0.47, 0.89]) and after (Adjusted OR: 0.64 [0.42, 0.96]) adjustment.

Compared to refugees resettled in the Northeast region of the United States, refugees resettled in the West were much less likely to have an inadequate USC (Crude OR: 0.56 [0.42, 0.75]); Adjusted OR: 0.58 [0.42, 0.79]). Refugees resettled in the South or Midwest did not have a significant difference in reporting an inadequate USC compared to refugees in the Northeast. Refugees resettled during 2011–2014 showed no statistically significant difference in having an inadequate USC compared to refugees resettled in 2015.

Both before and after adjustment, refugees with an education level greater than secondary education were 35% less likely to have an inadequate USC compared to refugees with a primary education (Crude OR: 0.65 [0.47, 0.90]; Adjusted OR: 0.65 [0.45, 0.93]). Refugees with no education, a secondary education, or an education described as other prior to resettlement had no statistically significant difference in reporting an inadequate USC compared to refugees with a primary education. Refugees with interrupted healthcare coverage were more likely to report an inadequate USC compared to refugees with uninterrupted coverage (Crude OR: 1.90 [1.57, 2.31]; Adjustment OR: 1.77 [1.43, 2.19]). Refugees with poor or no English proficiency had no statistically significant difference in reporting an inadequate USC compared to refugees with good English proficiency.

The results of the multinomial logistic regression models for the trichotomous USC outcome variables can be found in Table 3. Similar to the primary inadequate USC analysis (Table 2), refugees 10–19 years old were much less likely to have a severely deficient USC compared to refugees 20–49 years old before and after adjustment (Crude OR: 0.47 [0.32, 0.70]; Adjusted OR: 0.45 [0.27, 0.73]). Before adjustment, the oldest grouping—65–75 years old—were less likely to have a severely deficient USC compared to middle-aged refugees (Crude OR: 0.29 [0.15, 0.55]). Nevertheless, this association was not found to be statistically significant following adjustment (Adjusted OR: 0.50 [0.25, 1.01]). Refugees resettled into the South were more likely to have a severely deficient USC compared to refugees resettled into the Northeast (Crude OR: 1.53 [1.11, 2.11]). After covariate adjustment, this finding was not statistically significant (Adjusted OR: 1.31 [0.92, 1.86]). Like the primary USC analysis, refugees resettled into the West were less likely to have a severely deficient USC compared to refugees resettled into the Northeast (Crude OR: 0.69 [0.49, 0.97]). Nevertheless, the finding was not statistically significant after adjustment (Adjusted OR: 0.73 [0.50, 1.05]). Following adjustment, refugees resettled during 2011 were less likely to have a severely deficient USC compared to refugees resettled during 2015 (Adjusted USC: 0.61 [0.42, 0.89]). Refugees with a higher than secondary education level prior to resettlement were less likely to have a severely deficient USC compared to refugees with a primary education (Crude OR: 0.68 [0.48, 0.97]; Adjusted OR: 0.60 [0.40, 0.90]). Refugees with interrupted healthcare coverage were 2.28 times more likely to have a severely deficient USC compared to refugees with uninterrupted healthcare coverage (Crude OR: 2.72 [2.18, 3.38]; Adjusted OR: 2.28 [1.80, 2.89]). Surprisingly, refugees with no English proficiency were less likely to have a severely deficient USC compared to refugees with good English proficiency (Crude OR: 0.69 [0.50, 0.94]; Adjusted OR: 0.65 [0.43, 0.97]).

## 4. Discussion

In this study, we found that interrupted healthcare coverage was associated with a higher risk of having an inadequate USC. In contrast, young age (10–19 years old), resettlement into the West, and higher than secondary education prior to resettlement were associated with a lower risk. When inadequate USCs were further broken down into the severely deficient USC and deficient USC sub-groupings, a few significant results emerged. Refugees with an education level higher than secondary had a significantly lower likelihood of having a severely deficient USC, while refugees with interrupted healthcare were over twice as likely to have a severely deficient USC.

Limited information about the association between age and USC is known. In one study, participants who reported non-emergency-care-based USCs were generally older [12]. The unadjusted findings for the 65–75-year-old refugees in our study supported this finding, although not upon adjustment. One plausible explanation for the lower risk of inadequate USCs among 10–19 and 65–75 year olds is the additional support programs available to teenagers and the elderly. State and federal programs such as the Children’s Health Insurance Program (CHIP) and Temporary Assistance for Needy Families (TANF) may provide enough assistance to prevent many children from having an inadequate USC. Medicare may have a comparable effect on the elderly.

We are aware of only one study considering the effect of US region on USC. In the study, children living in the Midwest, South, and West regions of the United States were more likely to report a clinic as their USC compared to children from the Northeast [15]. How these findings relate to the findings of our study is unclear. Furthermore, the reason for the relatively low risk of inadequate USCs among refugees resettled in the West is not clear. Based on the results of the trichotomous USC outcome analysis (Table 3), the relatively low inadequate USC risk among refugees in the West is more due to a lack of deficient USCs (i.e., folk healer use or other healthcare sources) than a lack of severely deficient USCs. The higher risk of a severely deficient USC among refugees resettled in the South is consistent with known health access disparities in the South [16]. The relatively higher prevalence of interrupted healthcare coverage among refugees resettled in the South could partially lead to a decrease in dependable primary care use and/or an increase in emergency department dependence [8]. Nevertheless, this finding was not statistically significant after adjustment, and no difference in USC was found in the primary inadequate USC analysis for refugees resettled in the South (Table 2).

Few studies have considered the association between year of refugee resettlement and USC. A decreased likelihood of having an inadequate USC over time was expected considering the anticipated healthcare access benefits among refugees following the implementation of the Affordable Care Act. From 2010, when the Affordable Care Act was signed into law, an expectation of improved access for refugees over time is reasonable. Nevertheless, no difference in USC adequacy was found based on resettlement year in the primary analysis. In the sub-group analysis, refugees resettled in 2011 were less likely to have a severely deficient USC but much more likely to have a deficient USC when compared to refugees resettled in 2015. The effect of resettlement duration on USC is certainly worth our consideration, as USC practices and access opportunities may change over time. For instance, one prior study showed a correlation between length of time since resettlement and emergency department dependence [4]. The higher prevalence of severely deficient USC among refugees resettled in 2011 may be due to diminishing governmental assistance as the duration of resettlement increases. Unfortunately, due to the cross-sectional nature of the ASR data, evaluating the effect of time since resettlement on healthcare access was not possible in this study.

Regarding education level, one prior study showed a positive association between higher education level and having a non-emergency-care-based USC [12]. Our finding of refugees with a higher than secondary education being less likely to report an inadequate USC may support this finding. The broad knowledge, universal communication skills, and various social benefits often obtained from a higher level of education may better prepare well-educated refugees to navigate the complex US healthcare system. Nevertheless, an incremental decrease in risk of having an inadequate USC with increasing prior education level was not observed. To note, based on the sub-group analysis (Table 3), the lower likelihood of an inadequate USC among the highly educated seems predominantly secondary to the lower likelihood of severely deficient USCs among this refugee grouping. Therefore, emergency department dependence and complete lack of USC was significantly less common among the highly educated.

A higher risk of an inadequate USC among refugees with interrupted healthcare coverage is not surprising. In a prior study, participants with health insurance were more likely to report an adequate USC than uninsured participants [6]. The mentioned study results are supported by our inadequate USC and severely deficient USC results for refugees with interrupted healthcare coverage. Our findings support the common claim of insurance mandate supporters, who associate insurance coverage with a lower risk of inadequate USC dependence and improved healthcare access overall.

## 5. Limitations

This analysis has multiple limitations, some of which are mentioned in our previous interrupted healthcare manuscript [8]. First, ASR surveys have had high survey nonresponse rates. A response rate of 24 percent was achieved in the 2016 ASR. Although a low response rate was anticipated and accounted for in the original survey design, different response rates across demographics may have negatively affected the sample representation of the actual refugee population within the US. A helpful nonresponse analysis can be found in the Urban Institute’s 2016 ASR Annual Survey of Refugee Data File User’s Guide [14]. Second, of the 4037 eligible refugees, 32% could not or chose not to provide an appropriate answer to the USC question. Imputation was not used. Thus, approximately one-third of the eligible refugees were excluded from the final USC analysis. This exclusion could have skewed the results of the analysis.

## 6. Conclusions

To our knowledge, sociodemographic predictors of the USCs of refugees living in the US have not been comprehensively analyzed using credible data from a national sample of refugees. In this study, we further examined potential sociodemographic predictors of inadequate USCs—a key component of healthcare access—among refugees living in the US using national data from the 2016 Annual Survey of Refugees (ASR). Combining this data with our previous interrupted healthcare coverage findings provided a more comprehensive understanding of sociodemographic predictors of poor healthcare access among US refugees. Together, the findings may propel early refugee contacts toward more effective healthcare resource allocation and assist policy makers as they update programs that affect refugee healthcare access.

Since the completion of this analysis, the survey data for the 2017 and 2018 ASR surveys were released to the public. Additionally, the US Department of Health and Human Services recently announced a redesign for the 2021 ASR with the goal of improving survey quality and relevance for key stakeholders. The new data will provide opportunity for more robust analyses of refugee resettlement in the US [8].

Nevertheless, further strengthening of US refugee health information systems is needed to better understand and appropriately address US refugees’ unique and challenging circumstances and needs. Further refugee health data collection—especially longitudinal data collection to understand time-sensitive needs throughout the resettlement process—should be prioritized in future federal and state strategic plans.

## Figures and Tables

**Table 1:** Sociodemographic characteristics of study population by type of Usual Source of Care (USC).

Sociodemographic Characteristics	Total	Inadequate USC	Adequate USC	
	*n* (%) *	*n* (%) *	*n* (%) *	*p*-Value
**Gender**				0.0049
Male	1567 (53.66)	702 (48.15)	865 (51.85)	
Female	1362 (46.34)	528 (41.59)	834 (58.41)	
**Age (years)**				
10–19	269 (10.42)	99 (36.32)	170 (63.68)	0.0093
20–49	2045 (71.81)	901 (46.97)	1144 (53.03)	
50–64	405 (12.32)	157 (45.14)	248 (54.86)	
65–75	171 (5.44)	50 (35.64)	121 (64.36)	
**Resettlement Region (US)**				
Northeast	465 (16.50)	210 (48.75)	255 (51.25)	<0.0001
South	871 (32.09)	433 (51.94)	438 (48.06)	
Midwest	845 (25.65)	327 (44.81)	518 (55.19)	
West	735 (25.76)	253 (34. 90)	482 (65.10)	
**Resettlement Year**				0.0681
2011	389 (21.02)	188 (51.16)	201 (48.84)	
2012	664 (21.17)	282 (44.98)	382 (55.02)	
2013	488 (21.71)	184 (42.57)	304 (57.43)	
2014	622 (19.77)	255 (41.58)	367 (58.42)	
2015	739 (16.32)	306 (44.88)	433 (55.12)	
**Chronic Debilitating Condition**				0.2504
Yes	632 (19.69)	242 (42.53)	390 (57.47)	
No	2285 (80.30)	983 (45.78)	1302 (54.22)	
**Education Level before Resettlement**				0.0018
None	604 (27.00)	272 (48.85)	332 (53.15)	
Primary	699 (23.71)	265 (43.09)	434 (56.91)	
Secondary	797 (26.67)	350 (46.20)	447 (53.80)	
Higher	401 (10.58)	141 (33.12)	260 (66.88)	
Other	377 (12.04)	175 (51.55)	202 (48.45)	
**Healthcare Coverage Status**				<0.0001
Uninterrupted Coverage	1768 (59.17)	619 (38.27)	1149 (61.73)	
Interrupted Coverage	1042 (40.83)	556 (54.14)	486 (45.86)	
**Current English Proficiency**				0.2153
Good	1601 (52.82)	668 (43.47)	933 (56.53)	
Poor	857 (30.94)	369 (48.11)	488 (51.89)	
None	464 (16.24)	191 (45.13)	273 (54.87)	
**Citizenship**				<0.0001
Bhutan	91 (2.98)	43 (48.24)	48 (51.76)	
Burma	184 (10.21)	130 (69.65)	54 (30.35)	
Cuba	243 (5.85)	99 (41.22)	144 (58.78)	
Congo	109 (6.62)	64 (53.20)	45 (46.80)	
Iran	117 (5.72)	27 (20.11)	90 (79.89)	
Iraq	1054 (24.48)	318 (29.74)	736 (70.26)	
Somalia	166 (8.46)	46 (28.30)	120 (71.70)	
United States	210 (13.32)	95 (48.63)	115 (51.37)	
Other	348 (13.22)	178 (55.88)	170 (44.12)	
None	282 (9.16)	184 (67.58)	98 (32.42)	
**Current Number of Jobs**				0.2194
None	1283 (43.73)	496 (43.10)	787 (56.90)	
One	1550 (53.55)	691 (46.55)	859 (53.45)	
More than One	90 (2.71)	41 (50.73)	49 (49.27)	
**Marriage Status**				0.8697
Currently Married	1806 (59.39)	746 (44.93)	1060 (55.07)	
Not Currently Married	1118 (40.61)	482 (45.32)	636 (54.68)	

* *n* is number of unweighted participants, % is weighted according to the ASR person-level analytic weights.

**Table 2:** Association between sociodemographic characteristics and inadequate Usual Sources of Healthcare (USC).

Sociodemographic Characteristics	Inadequate USC vs. Adequate USC	
	Odds Ratio (95% Confidence Interval)	
	Crude	Adjusted
**Gender**		
Male	1.29 (1.03, 1.62)	1.23 (0.99, 1.52)
Female	1 (ref)	–
**Age (years)**		
10–19	0.64 (0.47, 0.89)	0.64 (0.42, 0.96)
20–49	1 (ref)	–
50–64	0.93 (0.71, 1.22)	1.07 (0.78, 1.47)
65–75	0.63 (0.41, 0.96)	0.71 (0.43, 1.18)
**Resettlement Region (US)**		
Northeast	1 (ref)	–
South	1.14 (0.86, 1.50)	0.99 (0.73, 1.34)
Midwest	0.85 (0.65, 1.20)	0.82 (0.61, 1.11)
West	0.56 (0.42, 0.75)	0.58 (0.42, 0.79)
**Resettlement Year**		
2011	1.29 (0.96, 1.73)	1.05 (0.76, 1.44)
2012	1.00 (0.77, 1.31)	0.91 (0.69, 1.22)
2013	0.91 (0.69, 1.21)	0.86 (0.64, 1.15)
2014	0.87 (0.66, 1.15)	0.79 (0.59, 1.06)
2015	1 (ref)	–
**Chronic Debilitating Condition**		
Yes	0.88 (0.70, 1.10)	0.93 (0.72, 1.21)
No	1 (ref)	–
**Educational Level before Resettlement**		
None	1.16 (0.89, 1.53)	1.23 (0.91, 1.67)
Primary	1 (ref)	–
Secondary	1.13 (0.87, 1.47)	1.02 (0.76, 1.37)
Higher	0.65 (0.47, 0.90)	0.65 (0.45, 0.93)
Other	1.41 (1.02, 1.94)	1.35 (0.96, 1.91)
**Healthcare Coverage Status**		
Uninterrupted Coverage	1 (ref)	–
Interrupted Coverage	1.90 (1.57, 2.31)	1.77 (1.43, 2.19)
**Current English Proficiency**		
Good	1 (ref)	–
Poor	1.21 (0.98, 1.49)	1.02 (0.79, 1.30)
None	1.07 (0.82, 1.39)	0.88 (0.62, 1.24)
**Current Number of Jobs**		
None	0.87 (0.72, 1.05)	1.23 (0.96, 1.57)
One	1 (ref)	–
More than One	1.18 (0.71, 1.98)	1.26 (0.69, 2.32)
**Marriage Status**		
Currently Married	1 (ref)	–
Not Currently Married	1.02 (0.84, 1.23)	1.09 (0.87, 1.37)

**Table 3:** Associations between sociodemographic characteristics and trichotomous Usual Source of Healthcare (USC) outcome.

Sociodemographic Characteristics	Severely Deficient USC vs. Adequate USC		Deficient USC vs. Adequate USC	
	Odds Ratio (95% Confidence Interval)		Odds Ratio (95% Confidence Interval)	
	Crude	Adjusted	Crude	Adjusted
**Gender**				
Male	1.51 (1.22, 1.86)	1.26 (0.98, 1.61)	0.96 (0.74, 1.26)	1.24 (0.84, 1.55)
Female	1 (ref)	–	–	–
**Age (years)**				
10–19	0.47 (0.32, 0.70)	0.45 (0.27, 0.73)	1.10 (0.72, 1.68)	1.36 (0.75, 2.46)
20–49	1 (ref)	–	–	–
50–64	0.87 (0.64, 1.18)	1.27 (0.89, 1.81)	1.09 (0.71, 1.65)	0.73 (0.44, 1.19)
65–75	0.29 (0.15, 0.55)	0.50 (0.25, 1.01)	1.52 (0.92, 2.50)	0.85 (0.46, 1.58)
**Resettlement Region (US)**				
Northeast	1 (ref)	–	–	–
South	1.53 (1.11, 2.11)	1.31 (0.92, 1.86)	0.63 (0.43, 0.92)	0.61 (0.39, 0.95)
Midwest	0.98 (0.70, 1.37)	0.94 (0.65, 1.35)	0.69 (0.47, 0.99)	0.73 (0.49, 1.09)
West	0.69 (0.49, 0.97)	0.73 (0.50, 1.05)	0.40 (0.26, 0.60)	0.41 (0.26, 0.63)
**Resettlement Year**				
2011	0.89 (0.62, 1.26)	0.61 (0.42, 0.89)	2.54 (1.74, 3.71)	2.86 (1.88, 4.36)
2012	0.89 (0.65, 1.20)	0.75 (0.54, 1.05)	1.38 (0.95, 1.99)	1.53 (1.01, 2.31)
2013	0.91 (0.67, 1.24)	0.78 (0.56, 1.09)	0.92 (0.59, 1.42)	1.12 (0.71, 1.79)
2014	0.88 (0.65, 1.19)	0.78 (0.56, 1.09)	0.86 (0.57, 1.30)	1.12 (0.71, 1.79)
2015	1 (ref)	–	–	–
**Chronic Debilitating Condition**				
Yes	0.64 (0.48, 0.83)	0.77 (0.57, 1.06)	1.50 (1.11, 2.03)	1.25 (0.86, 1.83)
No	1 (ref)	–	–	–
**Educational Level before Resettlement**				
None	0.93 (0.68, 1.28)	1.10 (0.78, 1.57)	1.73 (1.19, 2.52)	1.49 (0.97, 2.30)
Primary	1 (ref)	–	–	–
Secondary	1.30 (0.97, 1.73)	1.05 (0.76, 1.45)	0.74 (0.49, 1.13)	0.90 (0.56,1.45)
Higher	0.68 (0.48, 0.97)	0.60 (0.40, 0.90)	0.59 (0.34, 1.01)	0.80 (0.44, 1.45)
Other	1.20 (0.83, 1.73)	1.14 (0.76, 1.70)	1.91 (1.20, 3.02)	1.90 (1.14, 3.18)
**Healthcare Coverage Status**				
Uninterrupted Coverage	1 (ref)	–	–	–
Interrupted Coverage	2.72 (2.18, 3.38)	2.28 (1.80, 2.89)	0.83 (0.62, 1.12)	0.91 (0.64, 1.30)
**Current English Proficiency**				
Good	1 (ref)	–	–	–
Poor	1.12 (0.88, 1.41)	0.97 (0.73, 1.29)	1.47 (1.08, 1.99)	1.20 (0.82, 1.76)
None	0.69 (0.50, 0.94)	0.65 (0.43, 0.97)	2.20 (1.55, 3.12)	1.46 (0.89, 2.41)
**Number of Current Jobs**				
None	0.67 (0.54, 0.83)	1.16 (0.87, 1.55)	1.49 (1.14, 1.96)	1.41 (0.99, 2.02)
One	1 (ref)	–	–	–
More than One	1.16 (0.65, 2.06)	1.38 (0.72, 2.65)	1.25 (0.55, 2.83)	0.80 (0.31, 2.07)
**Marriage Status**				
Currently Married	1 (ref)	–	–	–
Not Currently Married	1.19 (0.96, 1.47)	1.23 (0.96, 1.58)	0.71 (0.54, 0.95)	0.75 (0.52, 1.10)

## Data Availability

The original data source can be found at the following DOI: https://doi.org/10.3886/E104642V4 (accessed on 29 July 2019). The entire SAS code used to obtain all statistical results can be obtained by request to the corresponding author.
